# Complete Recovery Following Intentional Potassium Cyanide Poisoning Despite Delayed Hydroxocobalamin Administration: A Case Report

**DOI:** 10.7759/cureus.114883

**Published:** 2026-08-20

**Authors:** Kinga Lichtarska, Maciej Łydka, Wiktoria Bajek, Joanna Falana, Justyna Lewandowska, Iga Machała, Natalia Libudzic, Julia Łaciak, Piotr Granatowski

**Affiliations:** 1 Internal Medicine, St. Elizabeth's Hospital, LUX MED Group, Warsaw, POL; 2 Internal Medicine, National Medical Institute of the Ministry of Interior and Administration, Warsaw, POL; 3 Internal Medicine, Independent Public Central Clinical Hospital in Warsaw, Warsaw, POL; 4 Internal Medicine, Warsaw Southern Hospital, Warsaw, POL; 5 Internal Medicine, Polish Red Cross Maritime Hospital, Gdynia, POL; 6 Internal Medicine, Ministry of the Interior and Administration Hospital, Krakow, POL; 7 Department of Medicine, Medical University of Silesia, Katowice, POL

**Keywords:** antidote, case report, critical care, cyanide poisoning, hemodialysis, hydroxocobalamin, metabolic acidosis, potassium cyanide, suicide attempt, supportive care

## Abstract

Cyanide poisoning is rare but is characterized by rapid clinical deterioration and a high risk of mortality. We present the case of a 25-year-old woman with suspected ingestion of up to 5 g of potassium cyanide purchased online in a suicide attempt. On admission, the patient was in critical condition, presenting with severe impairment of consciousness, respiratory failure, and circulatory shock. Hydroxocobalamin was administered approximately four hours after admission due to the need to obtain the antidote from another institution. Until specific treatment became available, intensive supportive therapy and stabilization of vital functions were provided.

Despite ingestion of a potentially lethal dose of cyanide and limited immediate availability of the antidote, complete clinical stabilization was achieved, and the patient was discharged in good general condition on hospital day 15 without permanent organ damage or neurological deficits. This case highlights the importance of early initiation of intensive supportive care to maintain vital functions and serve as a bridge until specific antidotal treatment can be administered.

## Introduction

Cyanide compounds occur both naturally and synthetically. Natural sources include cyanogenic plants such as apricot kernels and bitter almonds, while synthetic cyanides are widely used in industries such as metallurgy and chemical manufacturing [1-3]. Cyanide poisoning is a life-threatening condition due to its rapid onset and high risk of mortality [2,4]. The severity of symptoms and prognosis depend primarily on the dose, chemical form, and route of exposure. Exposure may occur through oral ingestion, inhalation, or, less commonly, through dermal absorption [4].

Cyanide exerts its toxic effects by binding to cytochrome c oxidase in mitochondria, leading to inhibition of oxidative phosphorylation and disruption of cellular respiration. Consequently, despite preserved oxygen transport from the lungs to tissues, cells lose the ability to utilize oxygen, resulting in histotoxic hypoxia. This causes a shift toward anaerobic metabolism with excessive lactate production and the development of severe lactic acidosis [1,2,4].

The organs most susceptible to hypoxia are the central nervous system and cardiovascular system due to their high metabolic demand. Clinical manifestations of acute cyanide poisoning initially include headache, dizziness, altered mental status, dyspnea, and tachycardia, progressing in severe cases to coma, seizures, respiratory failure, hypotension, shock, and cardiac arrest [1,2,4].

Cherry-red skin discoloration and a characteristic bitter almond odor may support the diagnosis of cyanide poisoning, although these findings are uncommon, occurring in approximately 11% and 15% of patients, respectively [5]. Importantly, their absence does not exclude cyanide poisoning [5].

Due to the rapidly progressive nature of cyanide poisoning, prompt recognition and immediate initiation of treatment are essential. Management includes oxygen therapy, stabilization of vital functions with supportive care, and administration of a specific antidote, such as hydroxocobalamin [1,2,4]. Hydroxocobalamin binds cyanide ions to form nontoxic cyanocobalamin, which is subsequently excreted in urine [4]. The recommended initial dose is 5 g, with an additional 5 g administered if clinically indicated [2,4]. Although cyanide exposure may occur accidentally or occupationally, intentional ingestion of cyanide salts is a recognized route of poisoning and has historically been associated with suicide [1]. Here, we report a case of severe suspected potassium cyanide poisoning in a 25-year-old woman who achieved a favorable clinical outcome despite ingestion of a potentially lethal dose and delayed administration of hydroxocobalamin.

## Case presentation

A 25-year-old woman with a history of mood disorder, treated on an outpatient basis with venlafaxine, mirtazapine, and lamotrigine and receiving psychotherapy, was found unconscious in her apartment by a friend with whom she had arranged to meet. The patient had been in contact with the friend approximately 2 hours and 45 minutes before she was found unconscious. The exact time of ingestion could not be established; however, based on the last confirmed contact, it was estimated to have occurred within the preceding 2 hours and 45 minutes. The friend subsequently contacted emergency medical services. The emergency medical team arrived approximately 11 minutes after the call, and the patient was admitted to the Toxicology Unit approximately 42 minutes later. An empty package labeled as containing 5 g of potassium cyanide and a suicide note were found next to the patient. As the product was not analytically verified and the exact amount ingested could not be independently confirmed, the exposure was considered a suspected ingestion of up to 5 g of potassium cyanide. Concomitant ingestion of her regularly prescribed medications could not be excluded because several antidepressant tablets were missing from their packages.

Upon admission to the Toxicology Unit, the patient was in critical condition. She was unconscious, with a Glasgow Coma Scale score of 6, and presented with inadequate spontaneous respiration, trismus, and cardiovascular instability. Oxygen saturation was approximately 92%, blood pressure was 75/45 mmHg, and heart rate was 120 beats/min. Her breathing was shallow and irregular. The pupils were initially dilated and later became mid-sized, with anisocoria (right greater than left). The skin was cool and moist, with preserved pink coloration despite severe cardiorespiratory compromise. No bitter almond odor was detected by the medical staff.

Arterial blood gas analysis performed on admission revealed profound metabolic acidosis, with a pH of 7.004, HCO₃⁻ of 8.2 mmol/L, and base excess of -23.0 mmol/L, accompanied by marked hyperlactatemia of 22.0 mmol/L. Serial arterial blood gas findings are presented in Table 1. Toxicological testing performed on admission demonstrated thiocyanate in the blood at a concentration of 0.66 mg/dL. Thiocyanate was measured using a colorimetric method; although this was not a reference analytical method, no alternative method was available at the treating institution. The laboratory report listed thiocyanate concentrations of >15.0 mg/dL as toxic and >20.0 mg/dL as lethal. Direct measurement of blood cyanide concentration was not performed because the reagents required for cyanide analysis were unavailable at the hospital laboratory. Given the high clinical suspicion of cyanide poisoning based on the circumstances of exposure and the clinical presentation, treatment decisions were based primarily on clinical findings rather than the measured thiocyanate concentration. Ethanol was not detected. Venlafaxine, mirtazapine, lamotrigine, and their metabolites were not specifically assessed; therefore, concomitant ingestion of these medications could not be excluded.

**Table 1:** Arterial blood gas analysis at selected time points

Parameter	Upon admission	Post-hemodialysis	5 hours post-hydroxocobalamin	Reference range
pH	7.004	7.260	7.338	7.350-7.450
HCO₃⁻ (mmol/L)	8.2	18.6	24.3	22-28
PCO_2_ (mmHg)	34.6	42.9	46.4	32.0-45.0
PO_2_ (mmHg)	80.7	279.0	183.0	75.0-100
Lactate (mmol/L)	22.0	6.3	2.0	0.5-1.6
Base excess (mmol/L)	-23.0	-7.8	-1.4	(-2.3)-(+2.3)

Laboratory findings are summarized in Table 2. Renal parameters indicated acute kidney injury, most likely secondary to hypotension and tissue hypoxia. Additional findings included mild rhabdomyolysis, hypermagnesemia, leukocytosis, an elevated C-reactive protein concentration, and a mild increase in aspartate aminotransferase (AST) activity. Alanine aminotransferase (ALT), coagulation parameters, blood glucose, sodium, potassium, and total calcium were within their respective reference ranges.

**Table 2:** Laboratory findings at selected time points *The laboratory report did not provide a reference range for blood thiocyanate; instead, it reported interpretive thresholds of >15.0 mg/dL as toxic and >20.0 mg/dL as lethal. ALT, alanine aminotransferase; AST, aspartate aminotransferase; INR, international normalized ratio; MCH, mean corpuscular hemoglobin; MCHC, mean corpuscular hemoglobin concentration; MCV, mean corpuscular volume; PT, prothrombin time; RBC, red blood cell count; WBC, white blood cell count

Parameter	Upon admission	Hospital day 2	Hospital day 7	Reference range
Hemoglobin (g/dL)	13.4	10.3	12.1	12.0-15.7
RBC (10⁶/µL)	4.31	3.31	3.94	3.93-5.22
Hematocrit (%)	42.7	30.9	35.5	34.1-44.9
MCV (fL)	99.1	93.4	90.1	79.4-94.8
MCH (pg)	31.1	31.1	30.7	25.6-32.2
MCHC (g/dL)	31.4	33.3	34.1	32.2-35.3
WBC (10³/µL)	28.62	9.64	4.42	4.23-10.00
Platelets (10³/µL)	331	131	497	140-400
Urea (mg/dL)	41.5	31.9	-	17.0-43.0
Creatinine (mg/dL)	1.72	0.47	0.69	0.60-0.90
Sodium (mmol/L)	142.7	140.3	135.2	136.0-147.0
Potassium (mmol/L)	4.26	3.73	4.59	3.50-5.10
Magnesium (mg/dL)	3.27	1.84	1.95	1.90-2.50
Total calcium (mmol/L)	2.40	1.93	2.20	2.10-2.62
AST (IU/L)	52	18	22	10-31
ALT (IU/L)	18	26	41	10-31
PT (s)	12.9	15.2	14.5	10.2-15.2
INR	1.16	1.39	1.32	0.8-1.2
Creatine kinase (U/L)	384	225	123	25-175
C-reactive protein (mg/L)	34.1	217.5	12.5	<5.0
Blood glucose (mg/dL)	97	159	108	70-100
Blood thiocyanates (mg/dL)	0.66	-	-	toxic: >15.0, lethal: >20.0*
Blood ethanol (g/L)	0.00	-	-	

Noncontrast computed tomography of the head performed during the first hospital day showed no acute abnormalities. Initial chest radiography demonstrated normal lung aeration, no evidence of pulmonary edema or focal inflammatory changes, and appropriate positioning of the endotracheal tube. Follow-up chest imaging performed on hospital day 6 showed an asymmetric reduction in transparency in the right perihilar region without other significant abnormalities.

Treatment and clinical course

Due to respiratory failure, the patient was intubated immediately upon admission, and mechanical ventilation in assisted mandatory ventilation (AMV) mode was initiated, with a positive end-expiratory pressure (PEEP) of 5 and 100% oxygen in the inspired gas mixture. Sedation with midazolam and propofol was started. In response to circulatory shock, continuous norepinephrine infusion and aggressive intravenous fluid resuscitation were administered. The key clinical events, therapeutic interventions, and outcomes are summarized chronologically in Table 3.

**Table 3:** Timeline of key clinical events, interventions, and outcomes

Time point	Key clinical event and management
Upon admission	The patient was in critical condition, with markedly impaired consciousness, respiratory and circulatory failure, hypotension, and severe metabolic acidosis with a lactate concentration of 22.0 mmol/L. She was immediately intubated and mechanically ventilated with 100% oxygen. Intravenous fluids, norepinephrine, sodium bicarbonate, and sedation were initiated.
Approximately 2 hours after admission	Hemodialysis was initiated as a supportive intervention for profound metabolic acidosis.
Approximately 4 hours after admission	After partial metabolic improvement during hemodialysis, with pH increasing to 7.26 and lactate decreasing to 6.3 mmol/L, the procedure was terminated approximately 10 minutes before its planned completion to allow prompt administration of hydroxocobalamin obtained from another institution. Hydroxocobalamin was administered at a dose of 5 g intravenously over 15 minutes.
Approximately 9-14 hours after admission	Metabolic acidosis resolved, and the lactate concentration decreased to 2.0 mmol/L. Progressive neurological improvement was observed, including reduced depth of coma, return of cough and swallowing reflexes, and recovery of motor responsiveness.
Hospital days 2-4	Renal function normalized. The patient developed fever and increasing inflammatory markers. Aspiration pneumonia was suspected, and empirical ceftriaxone was initiated, followed by the addition of amikacin because of persistent fever and elevated C-reactive protein levels.
Hospital day 7	Sedation was discontinued, and the patient was successfully extubated. Adequate spontaneous respiration was maintained, with oxygen saturation of 97-99%.
Hospital days 13-14	Electrocardiography showed sinus tachycardia and T-wave inversions in multiple leads, accompanied by a mildly elevated troponin I concentration. Subsequent echocardiography showed no significant abnormalities.
Hospital day 15	The patient was conscious, hemodynamically and respiratorily stable, and had no detectable neurological deficits or somatic complaints. She was transferred to an inpatient psychiatric unit for further treatment.

Intravenous sodium bicarbonate was administered for profound metabolic acidosis. Hemodialysis was initiated approximately two hours after admission as a supportive intervention for profound metabolic acidosis and was planned for a two-hour session. The procedure was terminated approximately 10 minutes before its planned completion following partial correction of metabolic acidosis (pH 7.26; HCO₃⁻ 18.6 mmol/L; lactate 6.3 mmol/L) to allow prompt administration of hydroxocobalamin obtained from another institution. Given the severity of the poisoning, the treating team prioritized prompt antidotal therapy over completion of the remaining dialysis time. A dose of 5 g of hydroxocobalamin was administered as a 15-minute intravenous infusion. Following administration of the antidote, the oxygen concentration in the inspired gas mixture was gradually reduced to 60%.

During hydroxocobalamin administration, transient bradycardia and premature ventricular contractions were observed. Shortly after completion of the infusion, characteristic red-orange discoloration of the skin and urine developed. Intensive supportive treatment was continued.

Over the following hours, the patient demonstrated progressive metabolic and neurological improvement. Approximately nine hours after admission, corresponding to five hours after hydroxocobalamin administration, metabolic acidosis had resolved, with near-normalization of the lactate concentration (pH 7.338, HCO₃⁻ 24.3 mmol/L, and lactate 2.0 mmol/L). After approximately 14 hours, the depth of coma decreased, cough and swallowing reflexes returned, and progressive recovery of motor responsiveness was observed. Concurrently, the previously observed anisocoria resolved, and the pupils became constricted and symmetrical. Renal function normalized by hospital day 2.

On hospital day 2, the patient developed a fever of up to 39°C accompanied by increasing inflammatory markers. Aspiration pneumonia was suspected as the source of infection, and empirical ceftriaxone therapy and antipyretic treatment were initiated. No microbiological investigations were performed. Because of persistent fever and a further increase in C-reactive protein levels, amikacin was added on hospital day 4. Follow-up chest radiography demonstrated decreased transparency in the right perihilar region, supporting the clinical suspicion of pneumonia. Ceftriaxone was administered for 10 days and amikacin for seven days.

Mechanical ventilation, sedation, antimicrobial therapy, fluid management, and temporary vasopressor support were continued during the following days. Vasopressor support was gradually reduced and discontinued after hemodynamic stability had been achieved.

On hospital day 7, sedation was gradually reduced and subsequently discontinued. The patient was successfully extubated and maintained adequate spontaneous respiration, with oxygen saturation of 97-99% during supplemental oxygen therapy. Transient post-extubation hoarseness and generalized weakness were managed conservatively, including inhaled budesonide.

During the following days, the patient progressively regained full consciousness and orientation. By hospital day 9, she was able to communicate appropriately and had no detectable focal neurological deficits. She remained hemodynamically and respiratorily stable, and the fever resolved.

Laboratory monitoring demonstrated transient mild liver injury, rhabdomyolysis, anemia, and thrombocytopenia, all of which gradually improved. Renal function remained normal after its initial recovery.

Following psychiatric evaluation on hospital day 12, continued inpatient psychiatric treatment was recommended. The patient’s pre-admission psychiatric medications had not been administered during hospitalization, as their exact doses and dosing schedule were initially unknown and were reported by the patient only after regaining consciousness. Modification of her psychiatric pharmacotherapy was planned in the inpatient psychiatric setting after resolution of the acute poisoning and stabilization of her somatic condition.

On hospital day 13, electrocardiography showed sinus tachycardia and T-wave inversions in multiple limb and precordial leads. Troponin I was mildly elevated. Echocardiography performed on hospital day 14 showed no significant abnormalities.

On hospital day 15, after complete somatic stabilization, the patient was discharged from the Toxicology Unit and transferred by medical transport to a psychiatric hospital. At the time of transfer, she was conscious, calm, hemodynamically and respiratorily stable, and reported no somatic symptoms.

## Discussion

In the present case, the patient was suspected to have ingested up to 5 g of potassium cyanide, based on an empty 5 g package found at the scene. This amount greatly exceeds values considered potentially lethal. The estimated fatal oral dose of potassium cyanide in adults is approximately 200-300 mg [6-8]. Blood cyanide concentrations of 0.5-1.0 mg/L are considered toxic, whereas levels of 2.5-3.0 mg/L are generally associated with fatal poisoning [6,9]. Cyanide is rapidly eliminated from the circulation, with an estimated blood half-life of approximately one hour [6]. The present case reflects the epidemiological pattern reported by Lee et al., as it also involved intentional oral ingestion of potassium cyanide in a suicide attempt. In their study of 255 fatal cyanide poisoning cases, 97.3% were related to suicide attempts and 98.8% resulted from oral exposure [10].

On admission, the clinical presentation was consistent with severe cyanide poisoning and included profound impairment of consciousness, circulatory shock, and severe metabolic acidosis with marked hyperlactatemia (22 mmol/L). Pink skin coloration was also observed despite severe cardiorespiratory compromise. Confirmatory measurement of blood cyanide concentration is generally not available rapidly enough to guide emergency management; therefore, diagnosis and treatment decisions are largely based on the exposure history, clinical presentation, and indirect findings [11]. Among these, marked hyperlactatemia may serve as a useful diagnostic and severity marker in acute cyanide poisoning [12,13]. In the present case, the markedly elevated lactate concentration further supported the diagnosis of severe cyanide poisoning.

Concomitant ingestion of the patient’s psychiatric medications could not be excluded because several tablets were missing from their packages. Some features of the initial presentation, including coma, hypotension, and tachycardia, could also occur in drug overdose. In addition, the presence of trismus raised the possibility of serotonin toxicity or another drug-related toxidrome. However, serum concentrations of the potentially co-ingested medications were not measured, and the available clinical data did not allow this differential diagnosis to be definitively confirmed or excluded. Nevertheless, the overall clinical picture, particularly the profound hyperlactatemia, severe metabolic acidosis, and circulatory compromise in the context of suspected cyanide exposure, was considered primarily consistent with severe cyanide poisoning.

Transient anisocoria was also observed on admission. Non-contrast head computed tomography revealed no structural intracranial abnormalities, and the anisocoria resolved concurrently with neurological improvement, when the pupils became constricted and symmetrical. As no specific cause was established, this finding could not be attributed to a particular toxic mechanism or medication.

Hydroxocobalamin was administered approximately four hours after admission because the antidote had to be obtained from another institution. The exact time of ingestion could not be established; based on the last confirmed contact with the patient, ingestion was estimated to have occurred within the 2 hours and 45 minutes preceding her discovery. Consequently, the precise interval between ingestion and antidote administration could not be determined. Given the rapidly progressive and potentially fatal nature of cyanide poisoning, early administration of specific antidotal therapy is recommended [11]. In the present case, the delay in access to hydroxocobalamin highlights the importance of immediate intensive supportive management while specific antidotal treatment is being obtained. Similar challenges related to antidote availability have been described by Vishnupriya et al., who reported survival of a 29-year-old patient after ingestion of approximately 8 g of potassium cyanide purchased online despite complete unavailability of antidotal therapy [8]. A comparable issue was reported by Namakizadeh Esfahani et al., in which recovery was achieved despite the lack of an available antidote, with treatment based on intensive supportive care and adjunctive plasmapheresis [1].

Alternative antidotal therapy with sodium nitrite and sodium thiosulfate was not administered because these agents were not available at the treating institution. Given their limited availability and less favorable safety profile compared with hydroxocobalamin, obtaining these agents from another institution was not considered a preferable alternative, and efforts were focused on obtaining hydroxocobalamin [14]. Hydroxocobalamin is currently regarded as the preferred antidote due to its rapid mechanism of action and favorable safety profile [3,9,15]. The recommended dose for adults is 5 g, administered as an intravenous infusion over 15-30 minutes; a second dose may be administered if clinically indicated [2,4,9]. In our patient, hydroxocobalamin administration was followed by the characteristic red-orange discoloration of the skin and urine [14,15]. Transient cardiac rhythm disturbances were also observed, although no severe complications occurred.

The transient electrocardiography abnormalities and mild troponin I elevation observed later in the hospital course were likely multifactorial. Potential contributing mechanisms included myocardial ischemia secondary to the initial circulatory shock, tissue hypoxia resulting from inhibition of cellular respiration, and direct cyanide-related cardiovascular toxicity. Subsequent echocardiography revealed no significant abnormalities, and the patient remained clinically stable, suggesting transient myocardial injury without persistent cardiac sequelae.

Hemodialysis is not considered a standard component of cyanide poisoning management, where treatment is primarily based on antidotal therapy and intensive supportive care [1]. In the present case, hemodialysis was initiated as supportive treatment for profound metabolic acidosis. The planned two-hour session was terminated approximately 10 minutes early to allow prompt administration of hydroxocobalamin. Hydroxocobalamin-induced discoloration may interfere with blood-leak detection systems in some hemodialysis machines and result in premature interruption of dialysis [14]. Given the severity of the poisoning, the treating team prioritized prompt antidotal therapy over completion of the remaining dialysis time. A rapid improvement in acid-base status and a marked reduction in lactate concentration were observed during this period. However, mechanical ventilation, fluid resuscitation, vasopressor support, sodium bicarbonate, and hemodialysis were initiated during the same early treatment period; therefore, the improvement in pH and lactate cannot be attributed to hemodialysis alone. Hemodialysis should therefore be regarded in this case as an adjunctive supportive intervention for severe metabolic derangement rather than as a specific treatment for cyanide poisoning.

This case also highlights the issue of limited antidote availability. Similar to the report by Vishnupriya et al. [8], delayed administration resulted from the lack of immediate access to hydroxocobalamin at the treating institution. These findings emphasize the need to improve logistical pathways and ensure timely access to antidotal therapies used in rare but potentially fatal poisonings. Hospital-based or regional stocks of hydroxocobalamin could provide earlier access to antidotal treatment, although their potential benefits should be weighed against the costs associated with maintaining stocks for a rare poisoning.

Several limitations should be acknowledged. The exact amount ingested could not be independently confirmed, and the composition of the product labeled as potassium cyanide was not analytically verified. The exact time of ingestion was also unknown. Direct blood cyanide measurement was unavailable because the required reagents were not available at the hospital laboratory. Thiocyanate was measured on admission using a colorimetric method that was not a reference analytical method; therefore, the measured concentration should be interpreted with caution. Diagnosis and therapeutic decisions were consequently based primarily on the circumstances of exposure and the clinical presentation. Finally, concomitant ingestion of psychiatric medications could not be definitively excluded because several tablets were missing and the concentrations of these medications were not specifically measured.

## Conclusions

The presented case highlights that rapid clinical assessment and immediate initiation of intensive supportive treatment are crucial in acute cyanide poisoning. Clinicians should consider cyanide poisoning in patients presenting with severe metabolic acidosis, altered consciousness, and unexplained respiratory or circulatory failure. Given the increasing availability of cyanide compounds through online sources, clinicians should remain aware of this potential route of exposure in patients presenting with suspected intentional poisoning. Despite suspected ingestion of a potentially lethal amount of potassium cyanide and delayed administration of hydroxocobalamin, the patient achieved a favorable clinical outcome with complete somatic stabilization by hospital day 15. Early intensive supportive treatment, including oxygen therapy, fluid resuscitation, vasopressor support, correction of severe metabolic acidosis, and close clinical and biochemical monitoring, contributed to maintaining vital functions until specific antidotal therapy became available. Hemodialysis was used as an adjunctive supportive intervention for profound metabolic acidosis; however, its independent contribution to the observed metabolic improvement cannot be determined because several therapeutic interventions were initiated concurrently.

This case also emphasizes the importance of organizational and preventive aspects in the management of severe poisonings. Limited availability of hydroxocobalamin and the resulting delay in antidote administration underline the need to optimize logistical procedures and ensure timely access to antidotal therapies used in life-threatening emergencies. Following intentional cyanide poisoning, comprehensive psychiatric evaluation and appropriate long-term mental health care remain essential.
